# Social and Behavioral Factors Associated with Lack of Intent to Receive COVID-19 Vaccine, Japan

**DOI:** 10.3201/eid2809.220300

**Published:** 2022-09

**Authors:** Takeshi Arashiro, Yuzo Arima, Ashley Stucky, Chris Smith, Martin Hibberd, Koya Ariyoshi, Motoi Suzuki

**Affiliations:** National Institute of Infectious Diseases, Tokyo, Japan (T. Arashiro, Y. Arima, A. Stucky, M. Suzuki);; London School of Hygiene and Tropical Medicine, London, UK (T. Arashiro, C. Smith, M. Hibberd);; Nagasaki University, Nagasaki, Japan (T. Arashiro, C. Smith, M. Hibberd, K. Ariyoshi, M. Suzuki)

**Keywords:** COVID-19, severe acute respiratory syndrome coronavirus 2, SARS-CoV-2, coronavirus disease, vaccine, vaccine hesitancy, viruses, respiratory infections, zoonoses, Japan

## Abstract

Persons in Japan who did not intend to receive COVID-19 vaccines after widespread rollout were less likely than others to engage in preventive measures or to be afraid of getting infected or infecting others. They were also not less likely to engage in potentially high-risk behaviors, suggesting similar or higher exposure risks.

COVID-19 vaccines have become a critical tool in pandemic control (*1*). In Japan, BNT162b2 (Pfizer-BioNTech, https://www.pfizer.com), mRNA-1273 (Moderna, https://www.modernatx.com), and ChAdOx1 nCoV-19 (AZD1222; Oxford/AstraZeneca, https://www.astrazeneca.com) have been approved, but use of ChAdOx1 nCoV-19 has been minimal. For the Omicron variant, 2 doses of mRNA vaccines might not be highly protective against symptomatic infection, but early data suggest they are still highly protective against severe disease and that a booster dose provides further protection (*2*–*4*). Addressing persons at highest risk for severe or fatal COVID-19 who do not intend to be vaccinated has become paramount as we transition to the endemic phase, which is especially true in Japan because most persons are not protected by natural infection (*5*). Several studies have addressed reasons behind this hesitancy at the early stage of vaccine rollout (*6*–*9*), but evidence on attitudes toward risk for infection and prevention and risk behaviors is scarce.

We retrospectively analyzed an online survey about life during the COVID-19 pandemic conducted by a marketing research company in Japan during November 26–28, 2021, after the vaccine rollout had stabilized and 70% of the population had received 2 doses. The total number of survey participants was 2,500 (250 participants for each sex and 10-year age group, 20–60 years of age) (Appendix). We extracted sociodemographic information, vaccination status (choices included vaccinated once, vaccinated twice, unvaccinated with intention to be vaccinated, unvaccinated without intention to be vaccinated, and prefer not to answer), attitudes toward COVID-19-related issues (e.g., whether participants were afraid of getting infected), and behaviors in the previous week (e.g., preventive measures such as mask-wearing and potentially high-risk behaviors such as visiting bars or restaurants) (*10*). For vaccination status, we categorized the first 3 options into vaccinated or intend to be vaccinated and the last 2 choices into no intention to be vaccinated, because persons who preferred not to answer likely did not intend to be vaccinated but were unwilling to disclose this information. Depending on the social or behavioral factor, we adjusted for potential confounders that were determined a priori (*6*–*9*). This study was reviewed and exempt from ethics approval by the Institutional Review Board of the National Institute of Infectious Diseases, Japan.

Overall, 2,069 (82.8%) participants had received 2 doses, 35 (1.4%) had received 1 dose, 95 (3.8%) were not vaccinated but intended to be, 203 (8.1%) had no intention of being vaccinated, and 98 (3.9%) preferred not to answer. By age group, proportions of vaccinated persons were similar to those in the general population of Japan (Appendix). The proportions of participants residing in each geographic region were also similar to the national distribution (Appendix). Compared with men 60–69 years of age, men 20–39 years of age, as well as women 20–40 years of age, were >2-fold more likely to have no intention of being vaccinated (Appendix Table 1). Persons who did not intend to be vaccinated were less likely to be afraid of getting infected (adjusted odds ratio [aOR] 2.32, 95% CI 1.53–3.53), family members getting infected (aOR 2.50, 95% CI 1.68–3.71), infecting others (aOR 2.58, 95% CI 1.73–3.84), and bed shortages caused by a surge in severe COVID-19 cases (aOR 1.89, 95% CI 1.25–2.87). Persons who did not intend to be vaccinated also did not plan to receive a third (booster) dose, but 74% of persons who had received or intended to receive vaccines also intended to receive a booster dose. Persons without intention to be vaccinated were more likely to report not wearing a mask (aOR 2.01, 95% CI 1.52–2.65) and not using hand sanitizer (aOR 1.90, 95% CI 1.47–2.47) in the previous week. These persons were less likely to have gone shopping for nonessential goods in the past week (aOR 0.70, 95% CI 0.51–0.97), but no association was seen between vaccination intent and refraining from meeting with others (aOR 1.20, 95% CI 0.87–1.65) or going to crowded places or traveling (aOR 1.11, 95% CI 0.83–1.47). We also saw no association between vaccination intent and meeting noncohabitating friends, acquaintances, or family members (aOR 0.73, 0.47–1.12); dining out (aOR 0.92, 0.65–1.30); going out socially (aOR 0.87, 0.59–1.27); traveling (aOR 0.51, 0.22–1.22); or going to a gym (aOR 1.08, 0.64–1.83). We obtained similar results when we excluded persons who preferred not to answer regarding their vaccination status.

Persons who did not intend to receive COVID-19 vaccines were less likely to engage in preventive measures or be afraid of getting infected or infecting others, but we observed no association between vaccine intention and engaging in potentially high-risk behaviors. These results suggest that these nonintenders have similar or higher exposure risks compared with vaccinees and intenders. Similar surveys might be considered in other countries to understand vaccine denial and inform policies and risk communication.

Limitations of our study include selection bias and recall bias. Social desirability bias might be an issue, but this survey about life during the pandemic was not administered as an assessment about COVID-19 vaccination intent.

Appendix Additional information about social and behavioral factors associated with lack of intent to receive COVID-19 vaccine, Japan
