# Supplementary material for: Social and Behavioral Factors Associated with Lack of Intent to Receive COVID-19 Vaccine, Japan
Source: Emerg Infect Dis. 2022 Sep;28(9):1909–10. doi: 10.3201/eid2809.220300 (PMC9423921; doi:10.3201/eid2809.220300)
Supplement: Appendix — Additional information about social and behavioral factors associated with lack of intent to receive COVID-19 vaccine, Japan [file 22-0300-Techapp-s1.pdf]

Article DOI: https://doi.org/10.3201/eid2809.220300

Social and Behavioral Factors Associated with Lack of Intent to Receive COVID-19 Vaccine, Japan

Appendix

Survey Background

The participants in the nationwide survey were those who voluntarily registered to be a panel member of a marketing research company. As of January 2022, the company had ≈5.41 million active panel members who have responded to ≥1 questionnaire in the past year (4.3% of 126.15 million population in Japan). In exchange for responding to questionnaires, panel members receive points that can be exchanged for products and services from partner companies.

The survey was conducted exclusively in Japanese. Although foreigners are an important consideration when formulating vaccination policy, they comprise only 2.2% (2.75 million out of 126.15 million) of the total population in Japan (*1*). The survey participants came from all 47 prefectures of Japan.

Comparison of Survey Participants and General Population of Japan

Since the survey was conducted on a voluntary basis, there was concern that the survey participants could differ from the general population of Japan. Therefore, we compared the survey participants and the general population for (1) proportion of persons vaccinated twice by age group and (2) geographic region of residence, in an attempt to evaluate the generalizability of our findings.

Data on the proportion of vaccinated persons in the general population by age group were only available for the date of accessing the data (i.e., February 14, 2022), but the national proportion of persons vaccinated twice has remained similar (73.0% on November 26, 2021, compared to 74.4% on February 14, 2022) (*2*). The proportion of individuals vaccinated twice among the survey participants was similar to the national distribution (Appendix Table 2). The proportions of participants residing in each geographic region were also similar to national proportions (Appendix Table 3).

Appendix Table 1. Association between lack of intention to receive COVID-19 vaccination and various social and behavioral factors based on a questionnaire conducted in late November 2021, Japan

Category	No intention to be vaccinated (n = 301)	Vaccinated or intend to be vaccinated (n = 2,199)	Crude odds ratio (95% CI)	Adjusted odds ratio (95% CI)
Age group, years/sex				
60–69/Male	16 (5.3)	234 (10.6)	1	-
50–59/Male	23 (7.6)	227 (10.3)	1.48 (0.76–2.88)	-
40–49/Male	27 (9.0)	223 (10.1)	1.77 (0.93–3.37)	-
30–39/Male	44 (14.6)	206 (9.4)	3.12 (1.71–5.70)	-
20–29/Male	43 (14.3)	207 (9.4)	3.04 (1.66–5.56)	-
60–69/Female	23 (7.6)	227 (10.3)	1.48 (0.76–2.88)	-

Category	No intention to be vaccinated (n = 301)	Vaccinated or intend to be vaccinated (n = 2,199)	Crude odds ratio (95% CI)	Adjusted odds ratio (95% CI)
50–59/Female	20 (6.6)	230 (10.5)	1.27 (0.64–2.52)	-
40–49/Female	36 (12.0)	214 (9.7)	2.46 (1.33–4.56)	-
30–39/Female	31 (10.3)	219 (10.0)	2.07 (1.10–3.89)	-
20–29/Female	38 (12.6)	212 (9.6)	2.62 (1.42–4.84)	-
Region of residence				
Hokkaido/Tohoku	36 (12.0)	259 (11.8)	1	-
Kanto	97 (32.2)	848 (38.6)	0.82 (0.55–1.24)	-
Chubu	60 (19.9)	347 (15.8)	1.24 (0.80–1.94)	-
Kinki	56 (18.6)	416 (18.9)	0.97 (0.62–1.51)	-
Chugoku/Shikoku	20 (6.6)	162 (7.4)	0.89 (0.50–1.59)	-
Kyushu	32 (10.6)	167 (7.6)	1.38 (0.82–2.31)	-
Marital status*				
Married, divorced, or widowed	130 (43.2)	1,353 (61.5)	1	1
Never married	171 (56.8)	846 (38.5)	2.10 (1.65–2.68)	1.85 (1.40–2.44)
Presence of children*				
Yes	84 (27.9)	1,036 (47.1)	1	1
No	217 (72.1)	1,163 (52.9)	2.30 (1.77–3.00)	2.01 (1.51–2.68)
Presence of cohabitants*				
Yes	203 (67.4)	1,730 (78.7)	1	1
No	98 (32.6)	469 (21.3)	1.78 (1.37–2.31)	1.66 (1.26–2.17)
Household income, Japanese Yen*				
4–10 million	60 (19.9)	832 (37.8)	1	1
>10 million	17 (5.7)	189 (8.6)	1.25 (0.71–2.19)	1.40 (0.79–2.48)
3–4 million	27 (9.0)	212 (9.6)	1.77 (1.09–2.85)	1.76 (1.08–2.86)
<3 million	80 (26.6)	406 (18.5)	2.73 (1.92–3.90)	2.90 (2.01–4.18)
Prefer not to answer	117 (38.9)	560 (25.5)	2.90 (2.08–4.03)	3.15 (2.24–4.41)
Occupation type*				
Business person (general)	71 (23.6)	604 (27.5)	1	1

Category	No intention to be vaccinated (n = 301)	Vaccinated or intend to be vaccinated (n = 2,199)	Crude odds ratio (95% CI)	Adjusted odds ratio (95% CI)
Business person (management)	5 (1.7)	143 (6.5)	0.30 (0.12–0.75)	0.33 (0.13–0.86)
Business person (executive)	5 (1.7)	40 (1.8)	1.06 (0.41–2.78)	1.45 (0.54–3.87)
Staff at public office or nonprofit organization, teacher	10 (3.3)	106 (4.8)	0.80 (0.40–1.61)	0.83 (0.41–1.68)
Contract worker	28 (9.3)	125 (5.7)	1.91 (1.18–3.07)	2.41 (1.47–3.95)
Self-employed (industry/commercial)	9 (3.0)	66 (3.0)	1.16 (0.55–2.43)	1.54 (0.72–3.29)
Self-employed (small office/home office)	3 (1.0)	8 (0.4)	3.19 (0.83–12.3)	4.15 (1.03–16.7)
Agriculture, forestry, fishery	1 (0.3)	9 (0.4)	0.95 (0.12–7.57)	1.19 (0.14–9.95)
Specialist (lawyer, healthcare worker)	6 (2.0)	72 (3.3)	0.71 (0.30–1.69)	0.77 (0.32–1.85)
Part-time employee	46 (15.3)	331 (15.1)	1.18 (0.80–1.75)	1.41 (0.92–2.16)
Homemaker	33 (11.0)	322 (14.6)	0.87 (0.56–1.35)	1.18 (0.72–1.94)
Student	17 (5.7)	78 (3.6)	1.85 (1.04–3.31)	1.61 (0.86–3.03)
Unemployed/retired	55 (18.3)	243 (11.1)	1.93 (1.31–2.82)	2.76 (1.83–4.16)
Other occupation	12 (4.0)	52 (2.4)	1.96 (1.00–3.85)	2.23 (1.12–4.42)
Being afraid of getting infected†				
Slightly to very afraid	263 (87.4)	2,082 (94.7)	1	1
Not at all	38 (12.6)	117 (5.3)	2.57 (1.74–3.79)	2.32 (1.53–3.53)
Being afraid of family members getting infected‡				
Slightly to very afraid	258 (85.7)	2,083 (94.7)	1	1
Not at all	43 (14.3)	116 (5.3)	2.99 (2.06–4.35)	2.50 (1.68–3.71)
Being afraid of infecting others†				
Slightly to very afraid	258 (85.7)	2,076 (94.4)	1	1
Not at all	43 (14.3)	123 (5.6)	2.81 (1.94–4.07)	2.58 (1.73–3.84)
Being afraid of bed shortages due to a surge in severe COVID-19 cases†				
Slightly to very afraid	263 (87.4)	2,077 (94.5)	1	1
Not at all	38 (12.6)	122 (5.6)	2.46 (1.67–3.62)	1.89 (1.25–2.87)

Category	No intention to be vaccinated (n = 301)	Vaccinated or intend to be vaccinated (n = 2,199)	Crude odds ratio (95% CI)	Adjusted odds ratio (95% CI)
Intention to receive COVID-19 booster vaccine (third dose)				
Yes	0 (0.0)	1,688 (76.8)	1	1
No	203 (67.4)	319 (14.5)	N/A	N/A
Prefer not to answer	98 (32.6)	192 (8.7)	N/A	N/A
Mask-wearing in the past week†				
Yes	188 (62.5)	1,759 (80.0)	1	1
No	113 (37.5)	440 (20.0)	2.40 (1.86–3.10)	2.01 (1.52–2.65)
Use of hand sanitizer in the past week†				
Yes	159 (52.8)	1,570 (71.4)	1	1
No	142 (47.2)	629 (28.6)	2.23 (1.75–2.85)	1.90 (1.47–2.47)
Handwashing with soap in the past week†				
Yes	135 (44.9)	1,370 (62.3)	1	1
No	166 (55.2)	829 (37.7)	2.03 (1.59–2.59)	1.88 (1.45–2.43)
Refrain from using public transport in the past week§				
Yes	60 (19.9)	457 (20.8)	1	1
No	241 (80.1)	1,742 (79.2)	1.05 (0.78–1.42)	1.05 (0.76–1.43)
Refrain from meeting others (including for work) in the past week†				
Yes	56 (18.6)	508 (23.1)	1	1
No	245 (81.4)	1,691 (76.9)	1.31 (0.97–1.79)	1.20 (0.87–1.65)
Refrain from going to crowded places such as events, traveling, or restaurants/bars in the past week‡				
Yes	78 (25.9)	654 (29.7)	1	1
No	223 (74.1)	1,545 (70.3)	1.21 (0.92–1.59)	1.11 (0.83–1.47)
Teleworking or remote learning in the past week†				
Yes	31 (10.3)	232 (10.6)	1	1
No	270 (89.7)	1,967 (89.5)	1.03 (0.69–1.53)	0.94 (0.61–1.45)
Gathering information about preventive measures against COVID-19 in the past week†				
Yes	55 (18.3)	626 (28.5)	1	1
No	246 (81.7)	1,573 (71.5)	1.78 (1.31–2.42)	1.56 (1.14–2.15)
Going shopping to buy food and essential goods in the past week‡				
No	101 (33.6)	611 (27.8)	1	1

Category	No intention to be vaccinated (n = 301)	Vaccinated or intend to be vaccinated (n = 2,199)	Crude odds ratio (95% CI)	Adjusted odds ratio (95% CI)
Yes	200 (66.5)	1,588 (72.2)	0.76 (0.59–0.98)	0.90 (0.69–1.19)
Going shopping to buy non-essential goods in the past week‡				
No	247 (82.1)	1,646 (74.9)	1	1
Yes	54 (17.9)	553 (25.2)	0.65 (0.48–0.89)	0.70 (0.51–0.97)
Meeting friends, acquaintances, or family members who live separately (noncohabitants) in the past week‡				
No	274 (91.0)	1,932 (87.9)	1	1
Yes	27 (9.0)	267 (12.1)	0.71 (0.47–1.08)	0.73 (0.47–1.12)
Going to work/school in the past week‡				
No	196 (65.1)	1,111 (50.5)	1	1
Yes	105 (34.9)	1,088 (49.5)	0.55 (0.43–0.70)	0.58 (0.43–0.79)
Going out to eat in the past week‡				
No	256 (85.1)	1,801 (81.9)	1	1
Yes	45 (15.0)	398 (18.1)	0.80 (0.57–1.11)	0.92 (0.65–1.30)
Going out socially in the past week‡				
No	264 (87.7)	1,902 (86.5)	1	1
Yes	37 (12.3)	297 (13.5)	0.90 (0.62–1.29)	0.87 (0.59–1.27)
Traveling in the past week†				
No	295 (98.0)	2,109 (95.9)	1	1
Yes	6 (2.0)	90 (4.1)	0.48 (0.21–1.10)	0.51 (0.22–1.22)
Going to a gym in the past week¶				
No	283 (94.0)	2,071 (94.2)	1	1
Yes	18 (6.0)	128 (5.8)	1.03 (0.62–1.71)	1.08 (0.64–1.83)
Going to yoga in the past week¶				
No	281 (93.4)	2,114 (96.1)	1	1
Yes	20 (6.6)	85 (3.9)	1.77 (1.07–2.93)	1.84 (1.10–3.11)

*Odds ratio adjusted for age group/sex and region.

†Odds ratio adjusted for age group/sex, region, marital status, presence of children, presence of cohabitants, household income, and occupation.

‡Odds ratio adjusted for age group/sex, region, marital status, presence of children, presence of cohabitants, and household income.

§Odds ratio adjusted for age group/sex, region, household income, and occupation.

¶Odds ratio adjusted for age group/sex, region, and household income.

Appendix Table 2. Proportions of individuals vaccinated twice among survey participants and the general population of Japan

Age group, years	Survey participants (%)	General population*
20–29	75.4	79.0
30–39	77.2	79.4
40–49	82.8	83.0
50–59	88.6	90.8
60–69	89.8	89.5

*Based on published data from the Vaccination Record System (*2*).

Appendix Table 3. Proportions of individuals residing in each geographic region among survey participants and the general population of Japan

Region of residence	Survey participants (%)	General population*
Hokkaido/Tohoku	11.8	10.9
Kanto	37.8	34.6
Chubu	16.3	16.8
Kinki	18.9	17.7
Chugoku/Shikoku	7.3	8.7
Kyushu	8.0	11.3

*Based on data from the 2020 Census (*1*).

References

1. 2020 Census: Outline of the results [in Japanese]. 2021 Nov 30 [cited 2022 Jun 5]. https://www.stat.go.jp/data/kokusei/2020/kekka/pdf/outline_01.pdf

2. Vaccination record system [in Japanese] [cited 2022 Jun 5]. https://www.kantei.go.jp/jp/content/nenreikaikyubetsu-vaccination_data.pdf
